# Porous ionic liquid polymer: A reusable adsorbent with broad operating pH range for speciation of nitrate and nitrite

**DOI:** 10.1038/s41598-019-47648-w

**Published:** 2019-07-31

**Authors:** Reyhaneh Nayebi, Ghazale Daneshvar Tarigh, Farzaneh Shemirani

**Affiliations:** Department of Analytical Chemistry, University College of Science, University of Tehran, P.O. Box 14155-6455, Tehran, Iran

**Keywords:** Natural hazards, Solid Earth sciences

## Abstract

Ionic liquids (ILs) are a class of fluids with unique physicochemical properties employing polymeric substances emerging new materials. Solidification of ILs into porous materials generates porous ionic liquid polymers that combine the unique characteristics of ILs with common porous materials and polymers. In this study, it’s the first time the porous ionic liquid polymer was chosen as a sorbent for extraction and speciation of nitrite and nitrate. Porous IL was prepared through polymerization of 1-allyl-3-methylimidazolium bromide monomers in the presence of azobisisobutyronitrile (AIBN) and crosslinking of ethylene glycol dimethyl acrylate (EGDMA). Parameters affecting the adsorbent performance were optimized. Under the optimal conditions, correlation coefficient (R^2^) was 0.9996 and LOD was 0.1 µg L^−1^. This method presented the linearity in the concentration range between 0.1–100 µg L^−1^ and the relative standard deviation was 3.2%. Finally, the adsorption behavior of the obtained sorbent for nitrate and nitrite determination in various real samples was evaluated. The result indicates that the porous ionic liquid polymer showed high adsorption capacity (233 mg g^−1^). The convenient preparation of the porous ionic liquid material, as well as high adsorption capacity for anionic pollutants predicted its broad application potential in anion removal materials.

## Introduction

Ionic liquids (ILs) are defined as inorganic/organic salts in the liquid state at the temperature below 100 °C^1^. Different organic cations and anions, resulting as a new class of non-molecular solvents with ionic characters are formed ILs^2^. Due to their unique properties, including negligible volatility, high ionic conductivity, chemical, and thermal stability, ILs are attracting interest in a wide variety of fields such as catalysis, synthesis, gas absorption solid phase extraction (SPE), and, liquid-liquid extraction (LLE)^3–6^.

Recently, researchers have attempted to extend simple methods that use ILs to replace traditional organic solvents^7^. For example, Ren *et al*. developed a strategy to synthesize acid anhydride modifier xylan in BMIM [Cl] IL, suggesting that ILs as a desirable alternative to conventional volatile organic solvents^8^. Also, ILs have been considered as an efficient coating material to separate and concentrate on various compounds and pollutants^9^. Despite their benefits, ILs cannot be directly used in solid phase extraction due to their liquidity and they should be immobilized on the solid matrices. Various studies have introduced solid matrices for ILs^10,11^. Immobilization of ILs on the solid matrices is not an easy step and sometimes solid matrices themselves are toxic^5^.

Lately, ILs have been used to modify or synthesis a variety of polymers^7^. Kim *et al*. synthesized different nano-sized conducting polymeric materials via self-assembly, using magnetic ionic liquid (BMIM [FeCl_4_]) as solvent^12^. A series of imidazolium iodide ionic liquids containing n-alkyl groups and polyethylene glycol were prepared by Ganapatibhotla *et al*.^13^.

On the other hand, ILs exhibit low chemical stability. Fortunately, the chemical stability can be improved by the polymerization of ILs, yielding polymeric ionic liquids (PILs)^14^. PILs are synthesized from the IL monomers or copolymerization of ILs with other monomers. They combine some unique properties of polymeric architectures and ILs. PILs presented some of the unique properties of ILs such as ionic conductivity, thermal and chemical stability^15^. Ohno and Ito reported the polymerization of vinyl imidazolium type IL with high conductivity^16^.

Some groups reported the application of PILs in coating different materials and producing new sorbents for the separation of pollutants including cellulose-Fe_3_O_4_-PIL for biosorption of Congo red dye^17^, Fe_3_O_4_@SiO_2_@PIL for Allura red separation^18^, and PILs/GO-Silica for phenolic acids extraction^19^.

Over the past years, on the basis of logical molecular design, tremendous porous material with accessible pore channels and high surface area have been reported, like metal-organic frameworks (MOF)^20^, porous organic polymers^21^, and porous aromatic frameworks (PAFs)^22^. Recently, some researchers suggested that the charged porous materials would bring about more benefits over the natural analogues. These benefits include that: 1- the charged skeleton of the porous materials can strengthen the host-guest attraction or repulsion that is useful for the application in selective separation, 2-counter ions of the charged pores can be easily exchanged for the adjustment of porosity, an increase of adsorption behaviors^21,23^. Therefore, the scope of the applications of charged porous materials can be further developed.

Solidification of ILs into porous materials generates porous ionic liquid polymers that combine the unique characteristics of ILs with usual porous materials and polymers. Porous ILs have enlarged surface area and pore volume, great chemical stability and charged pores for storage and separation of analytes^15^. The main advantages of porous ILs are their charged pores and solidity. The charged pores of porous ILs serve as the ion exchange materials for capturing ionic pollutants^24^. Also, these materials are solid and can be used as a sorbent in the SPE procedure and there is no need for any solid support matrix to immobilize ILs onto them. Porous ILs have excellent selectivity and sorption capacity due to the presence of some interactions (π–π stacking, hydrogen bonding, ion exchange interaction and electrostatic). Furthermore, porous ILs indicated good compatibility with solvents, high thermal stability, broad operating pH range and special reusability^25^. Recently, porous ILs have been synthesized and applied for the pollutant separations by some groups^26,27^. So, this compound seems to be an effective adsorbent for the extraction process.

Nitrate and nitrate are the common mineral ions in the environment. Nitrate enters our body in many ways, one of which is drinking water. The nitrate enters the saliva and then it is converted to nitrite. Thus, the knowledge on the speciation of nitrate and nitrite is important. Most of the speciation methods are based on the nitrate reduction. Among some reduction agent, zinc was a good alternative for the reduction of nitrate to nitrite^28^.

One of the ways in which nitrite is extracted from the sample solution is the electrostatic interaction between the adsorbent and anions. So, the adsorbent should have a positive charge. The pH_pzc_ (pH zero-point charge) of the most adsorbents used for the nitrate and nitrite extraction is lower than 7^29–32^. When pH < pH_pzc_, the surface of the desired adsorbent is positive. It should be noted that the pk_a_ of HNO_2_ is 3.3 and most of NO_2_ are protonated in the acidic medium. So, it is necessary to use the adsorbent which its surface charge does not change in various pH. As previously described, porous IL can be used in a wide range of pH and it seems to be a great adsorbent for the nitrite extraction.

**Figure 1:**
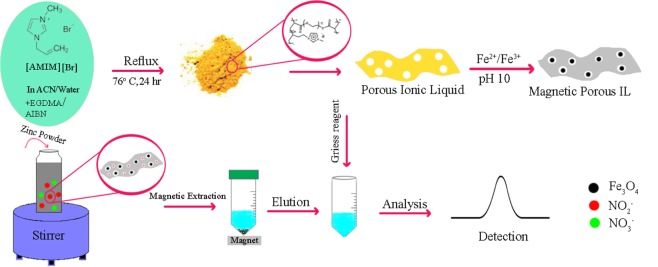
A schematic diagram for the synthesis of magnetic porous ionic liquid and representation of the DMSPE process.

Herein, we report an effective and easy strategy for the synthesis of porous IL and its application for nitrite absorption. This polymer was prepared via copolymerization of 1-allyl-3-methylimidazolium bromide [AMIM][Br] and crosslinking of ethylene glycol dimethyl acrylate (EGDMA). The type of solvent that is selected in the polymerization process is very important and affects the final product. When polymerization was done in the absence of a suitable solvent, no porous product could be obtained. To get to the desired product, the solvent with good solvency for both IL and EGDMA should be considered in the synthesis. Considering these conditions, an efficient adsorbent was synthesized and used for dispersive magnetic solid phase extraction (DMSPE) of nitrite (Fig. 1). The present study provides a new method for the direct use of ionic liquids in the extraction process.

## Experimental

### Reagents

All chemicals and reagents used were of analytical reagent grade, and double distilled water was used in the preparation of all solutions. 1-allyl-3-methylimidazolim bromide was purchased from Chemistry and chemical engineering research center (Karaj, Iran,). Azobisisobutyronitrile (AIBN), ethylene glycol dimethylacrylate (EGDMA), acetonitrile, sulfanilic acid, N-(1-Naphthyl) ethylene diamine (NED), ammonium ferric sulfate, ammonium ferrous sulfate, nitric acid, zinc powder, ammonium hydroxide and, hydrochloric acid were purchased from Merck (Darmstadt, Germany). Standard solutions were prepared by dissolving the appropriate amount of sodium nitrite and sodium nitrate in water and diluting to 100 mL. NED (0.1 g in 100 mL water) and sulfanilic acid (1 g in 100 mL phosphoric acid 5%) were used and stored in the darkness at 4 °C. The pH of solutions was adjusted by 0.5 mol L^−1^ NaOH and HCl solution.

### Equipment

UV-Vis spectrophotometer (Perkin Elmer, Lambda 25) was used to measure the absorbance of azo dye (www.perkinelmer.com). A Eurostar20 mechanical stirrer combining the glassware stirrer (IKa, UK) was applied for solution stir. The pH of aqueous solutions was measured by a digital pH meter (model 781) (Herisau, Switzerland) equipped with a glass combination electrode. Transmission electron microscopy (TEM) (Philips EM-400) (California, United States) and scanning electron microscopy (SEM) (TESCAN, MIRA III, Czech Republic) were employed to investigate the porous structure and surface morphology. X-Ray power diffraction (XRD) measurements were performed using a Philips PW1730 (Netherland) with monochromatized Cu-K_α_ radiation. The FT-IR spectra (400–4000 cm^−1^) were recorded using KBr pellets by Bruker, Tensor27 (USA).

### Preparation of porous ionic liquid polymer

Various solvents were chosen for the synthesis step including DMF (N, N-dimethylformamide), acetonitrile and acetonitrile/water. Among them, acetonitrile/water was an appropriate solvent for preparation of porous IL. So, [AMIM][Br] (2 mmol) were dissolved in 40 mL acetonitrile/water (4/1, V/V) followed by the addition of 0.15 g AIBN and 10 mmol EGDMA. After 3 h of stirring at room temperature, the mixture was refluxed at 76 °C for 24 h with stirring. The porous ionic liquid polymer in the form of yellow powder was collected and washed with water and ethanol and dried at 65 °C for 6 h.

### Preparation of magnetic porous ionic liquid polymer

To separate the adsorbent from the solution, magnetic nanoparticles were decorated on the adsorbent’s surface. Porous ILs are magnetized with paramagnetic particles^33^. The synthesized polymer was modified by Fe_3_O_4_ magnetic nanoparticles. Typically, 5 g of (NH_4_)_2_Fe(SO_4_)_2_·6H_2_O and 7.5 g of NH_4_Fe(SO_4_)_2_ were dissolved in the alkaline solution and then 3 g of the synthesized polymer was added to the iron solution under stirring. After 10 min, the mixture was sonicated at 80 °C for 1 h. The black precipitate was collected from the solution using a magnet. Separated magnetic porous IL (M-porous IL) was washed with deionized water three times and dried at 80 °C for 4 h.

### Sample preparation

Tap water sample was obtained after flowing 15 minutes in our laboratory.

Mineral water (Neptune Brand) was purchased from a local market in Tehran. Sampling of river water was conducted from the Savadkuh (Iran, Mazandaran). Fruits, grains, legumes and, nuts were purchased from a grocery. Fruits were cleaned, rinsed and stored in a freezer until analysis. For cooking samples, samples were cooked at 100 °C using distilled water without adding salt for 30 min. For bread, including both traditional and industrial, samples were dried at 60 °C for 12 h. For pistachio and almond, hard shells were removed, chopped and powdered using grinder mill. Sample preparation was conducted according to ISO method (ISO, 1984, 2004). Briefly, 1 g of the samples were treated with 5 mL sodium tetraborate solution in 100 mL hot water for 15 min. Afterward, 1 mL potassium hexacyanoferrate (II) trihydrate and 2 mL zinc acetate solution were added to obtain a clear liquid sample. Extraction was followed by filtration through filter paper (Whatman, 41, Ashless, 12.5 cm). The prepared solutions were assessed for nitrite and nitrate amount using the spectrophotometric method.

### General procedure

The magnetic porous IL-based DMSPE procedure was as follows: 2 mL of the magnetic porous IL suspension (including 5 mg of the adsorbent in deionized water) was dispersed into the 40 mL sample solution containing 20 μg L^−1^ of nitrite and the solution volume was adjusted at 50 mL using distilled water. The sample solution agitated for 5 min with mechanical stirrer equipped with glassware stirrer (1000 rpm). In this level, nitrite anions were electrostatically absorbed in the positive pores of the adsorbent. Then, an external magnetic field was used to separate the M-porous IL adsorbents from the solution. After a few seconds, the supernatant solution was completely decanted. 1 mL of nitric acid (1 mol L^−1^) as eluent solution was added to desorb the target analyte. The mixture was shaken up for 7 min and the magnetic adsorbent again exposed on the magnet. The eluent was injected into a quartz cell. Afterward, an equal volume of Griess reagent (0.1% NED and 1% sulfanilic acid in 5% H_3_PO_4_) was added and agitated for a few minutes at room temperature. The cell was placed in the spectrophotometer for measuring the absorbance at 540 nm.

In the presence of zinc powder and ammonia solution, nitrate is reduced to nitrite. For this aim, 2 drops of ammonia solution and 0.1 g (±0.1 mg) of zinc powder were added to the nitrite solution and incubated for 5 min. The nitrite in the solution plus the reduced nitrate was determined using Griess reagent. The amount of nitrite present in the real samples is determined without the reduction step. By deducting the total amount of nitrite after reduction and the initial nitrite concentration, concentration of nitrate was calculated.

## Results and Discussion

### Characterization of porous ionic liquid polymer

**Figure 2:**
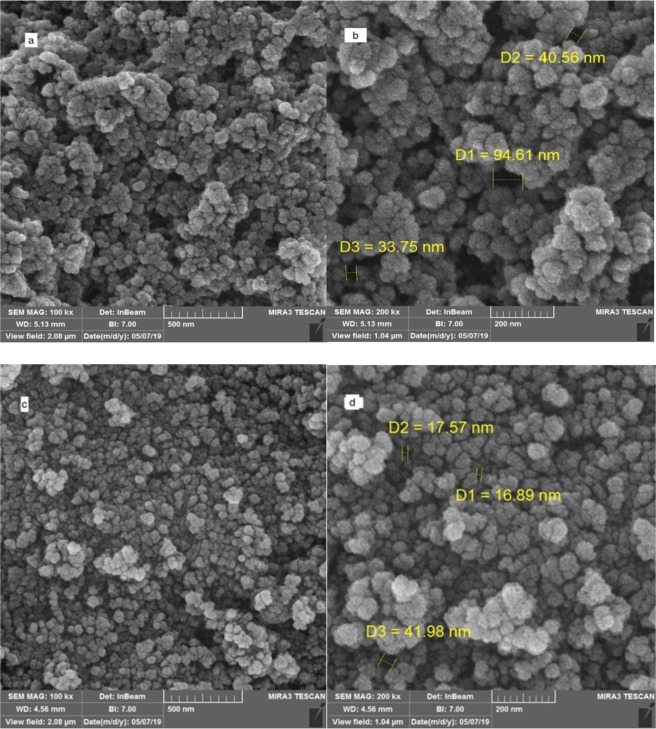
SEM image of the porous ionic liquid polymer (**a**,**b**), magnetic porous ionic liquid polymer (**d**,**c**).

**Figure 3:**
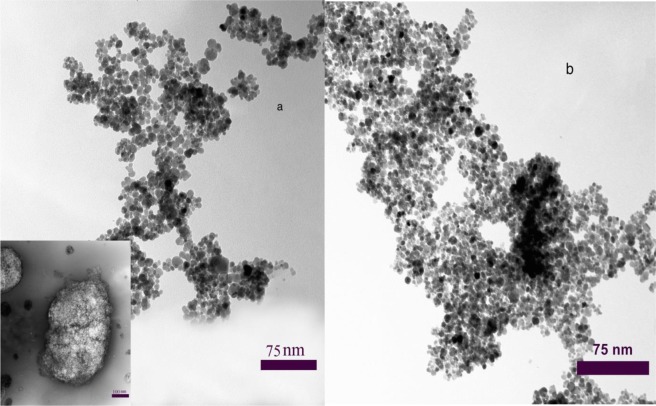
TEM image of the porous ionic liquid polymer, (inset: foam structure of porous IL) (**a**), magnetic porous ionic liquid polymer (**b**).

Surface morphology and the porous structure of the obtained polymer were investigated by SEM and TEM. The SEM images of the synthesized porous ionic liquid polymer (Fig. 2a) present a clear hierarchal porous structure. The particles are nano-sticks with size in the range of 30–95 nm, which may result from the different degree of polymerization (Fig. 2b). The particles are intertwined with each other to form a cross-linked framework and corresponding meso and macro porous structures that is further confirmed by TEM images. In the magnetic state, the pore size is reduced that clearly proves the presence of Fe_3_O_4_ nanoparticles in the cavities (Fig. 2d,c). In Fig. 3, can be clearly observed that the synthesized product is constructed by microspheres linked together. The TEM image exhibits many pores which were formed in the polymerization procedure. The major irregular particles with diameter ranging from decades of nanometers to hundreds of micrometers were shown in Fig. 3. These particles are rigidly interacted with each other and weakly packed into big aggregates, creating a foam structure. It was, in addition, validated that the porous ionic liquid polymer owned by hierarchical porous polymer, due to the coexisting of micro, meso and macro pores in the hierarchical porous polymer. Dark spots represent magnetite nanoparticles (Fig. 3b). By the way, the results have confirmed the successful performance of the synthesis procedure.

**Figure 4:**
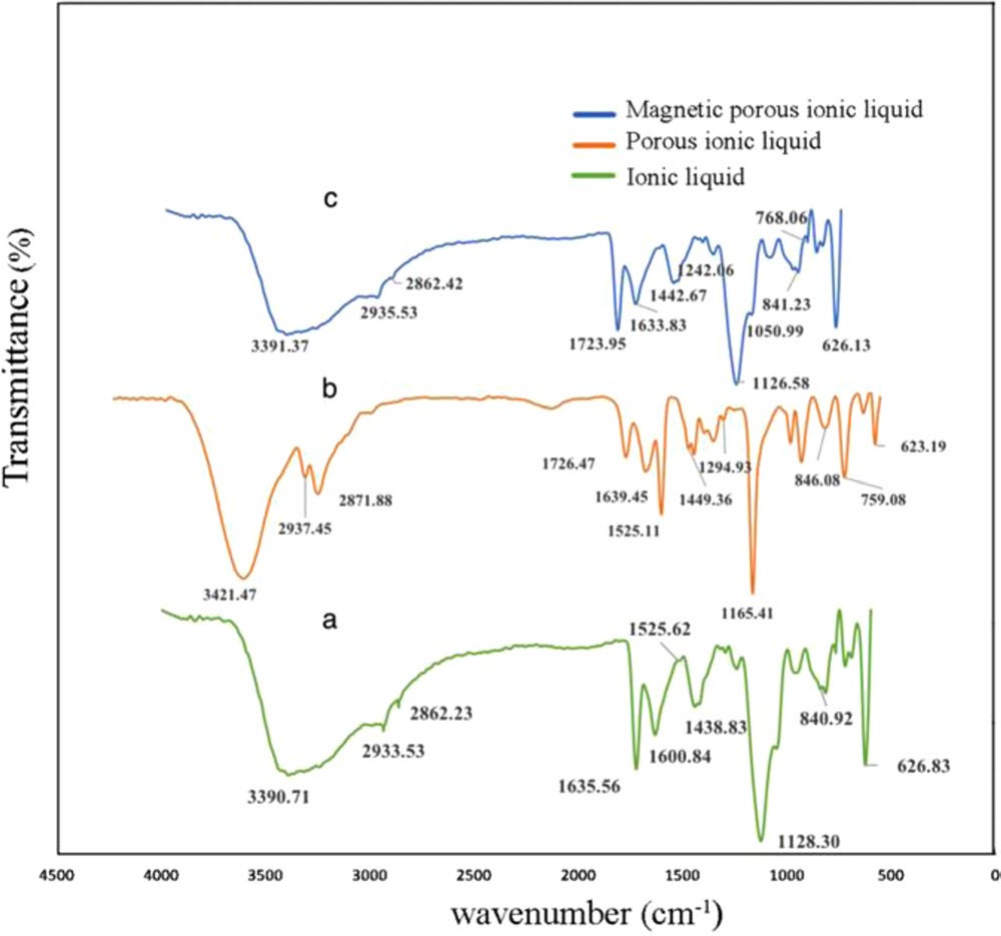
FT-IR spectra of the ionic liquid (**a**), porous ionic liquid polymer (**b**), and magnetic porous ionic liquid polymer (**c**).

Fourier transform infrared spectroscopy in wavenumber of 4000-400 cm^−1^ was used for recognition of characteristic functional groups of the synthesized polymer before and after polymerization and magnetic state. As shown in Fig. 4, the peaks at 2935 cm^−1^ and 2862 cm^−1^ are the aliphatic symmetric and asymmetric (C–H) stretching vibration due to the methyl groups. A broad absorption band at approximately 3100–3500 cm^−1^ is due to the quaternary amine salt formation with bromide. Wave numbers 1600 cm^−1^ and 1635 cm^−1^ are due to C=N and C=C stretching. A peak at 840 cm^−1^ is due to C–N stretching vibration. Specifically, the peaks at 1438 cm^−1^ and 1525 cm^−1^ are related to imidazolium group. In porous structure, the new absorption peaks of C=O stretching band at 1726 cm^−1^, C=C stretching bands at 1449 cm^−1^ and 1639 cm^−1^, C=N stretching at 1294 cm^−1^ and C–O–C stretching band 1165 cm^−1^ displayed that porous ionic liquid polymer was successfully prepared using [AMIM][Br] as monomer and EGDMA as cross linker. In the magnetic state, the peak at 626 cm^−1^ is due to Fe–O characteristic band and verified the synthesis of magnetic porous ionic liquid polymer.

**Figure 5:**
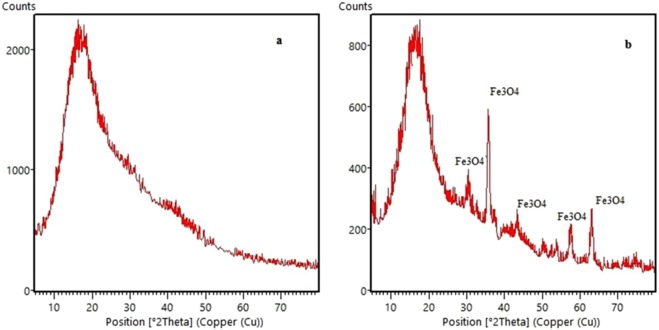
XRD patterns of porous ionic liquid polymer (**a**), magnetic porous ionic liquid polymer (**b**).

Figure 5 shows the XRD patterns of the obtained polymer. The peaks at 2θ = 30.28°, 35.69°, 43.38°, 53.69°, 57.49°, 63.01°, and 74.78° for cubic Fe_3_O_4_ (JCPDS no. 01-075-0449) are the characteristic peaks of the magnetic porous ionic liquid. The size of nanoparticle in the crystal structure of the synthesized polymer was estimated using Debye-Scherrer formula to be 12 nm at 2θ = 35–36° (main peak) for Fe_3_O_4_ and 6 nm at 2θ = 14–16° (main peak) for porous ionic liquid.

### Optimization of method

To achieve the optimal DMSPE extraction conditions, variables affecting the extraction performance were checked and optimized. One parameter was changed at one time to optimize the mentioned parameters. Each experiment was performed in triplicate. The following experimental conditions were found to give best results.

### Effect of pH

**Figure 6:**
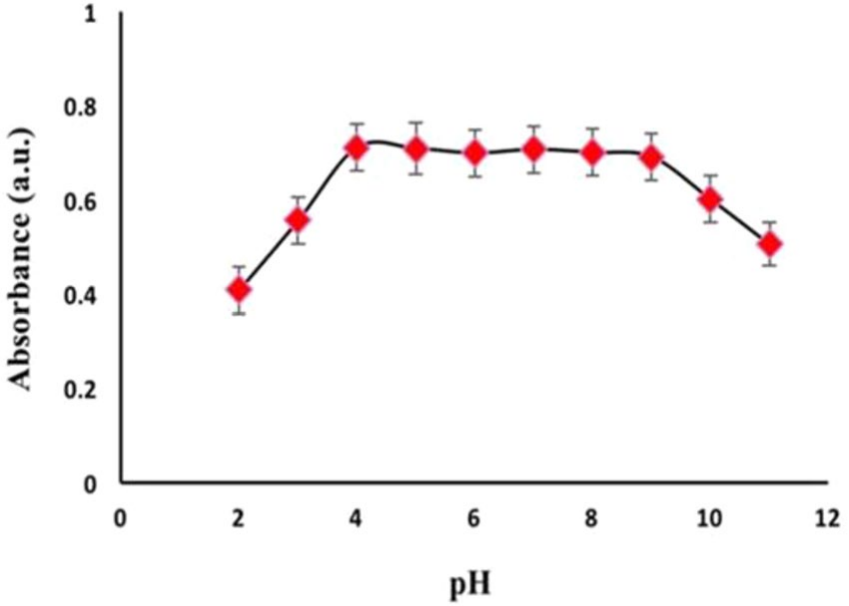
Effect of pH on absorbance. Concentration of NO_2_^−^ = 20 µg L^−1^, 10 mg M-porous IL, extraction time = 7 min, Elution with 1 M of HNO_3_.

The pH of sample solutions is an important parameter that affects the DMSPE efficiency. It can affect the adsorption sites of the adsorbent and make it passive or active. The effect of pH value on the sorption process was investigated ranging from 2–11. As shown in Fig. 6, sorption was enhanced with the increase of pH and it reached the highest at pH 4. With the increase of the pH value up to 9, extraction efficiency increased. By increasing the pH further, the extraction efficiency decreased. It can be explained that with the increase in pH value, imidazolium groups of the porous IL become more active and can participate in an anion exchange procedure that results in higher absorption of the nitrite ions. But, at highest pH, the competition between hydroxide and target anion for the same sorption site of porous IL, results in decreasing extraction efficiency.

### Effect of sorbent amount and sample volume

**Figure 7:**
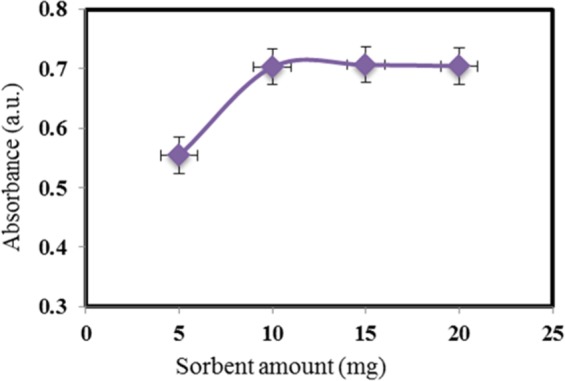
Effect of sorbent amount on absorbance intensity. Concentration of NO_2_^−^ = 20 µg L^−1^, 10 mg M-porous IL, extraction time = 7 min, Elution with 1 M of HNO_3_.

To acquire the least amount of the sorbent that demonstrates quantitative extraction of nitrite, 5–20 mg of the M-porous IL was added to the sample solution. As shown in Fig. 7, 10 mg (±0.1) is the best mass to quantitative sorption of the anions. This is caused by the high surface area to volume ratio of porous structures.

To achieve the possibility of enriching NO_2_^−^ from real samples with a good preconcentration factor, the performance in nitrite sorption from various sample volume ranging from 50–200 mL was tested. Results indicate that when the sample volume is less than 150 mL, nitrite ions were quantitatively recovered.

### Eluent type

**Figure 8:**
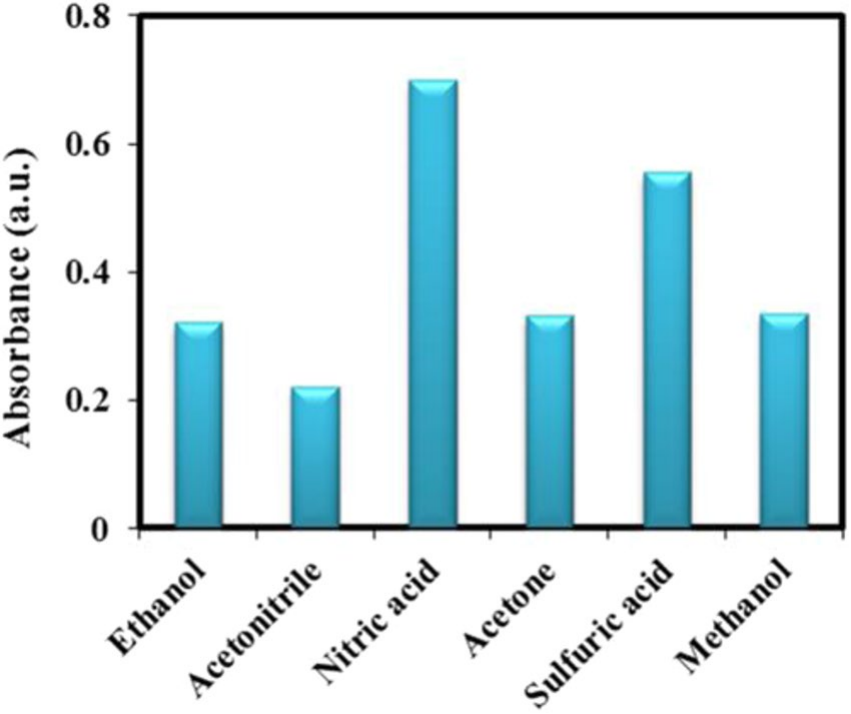
Effect of elution solvent on absorbance intensity. Concentration of NO_2_^−^ = 20 µg L^−1^, 10 mg M-porous IL, extraction time = 7 min, Elution with 1 M of HNO_3_.

Desorption of nitrite by the M-porous IL was investigated using various eluents, including methanol, ethanol, acetonitrile, nitric acid, sulfuric acid and, acetone. According to the elution efficiency, nitric acid (1 mol L^−1^) has the exceptional desorption ability in comparison of other eluents (Fig. 8). So, for further study, it was used as eluent.

For choosing the suitable volume of the eluent, a different volume of nitric acid was tested. Desorption was quantitative using o.4 mL of nitric acid.

### Elution time

**Figure 9:**
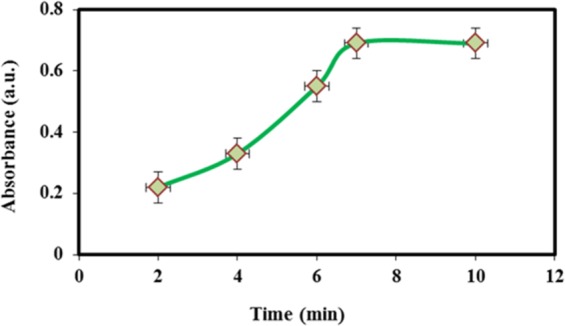
Effect of elution solvent on absorbance intensity. Concentration of NO_2_^−^ = 20 µg L^−1^, 10 mg M-porous IL, extraction time = 7 min, Elution with 1 M of HNO_3_.

The elution process was investigated in the range of 1–10 min; the elution efficiency did not change after 7 min (Fig. 9). It indicated that the nitrite could be desorbed quantitatively after 7 min elution and separated quickly due to the magnetic properties of the adsorbent.

### Sorption capacity

For equilibrium adsorption experiments, 2 mL of magnetic porous ionic liquid polymer solution (contains 0.50 g of the adsorbent) was dispersed in the standard solutions of NO_2_^−^ in the range of 1–70 ppm. For each one, the equilibrium concentration was calculated using the following equation:

$$Q=\frac{({C}_{0}\,\mbox{--}{C}_{e})V}{m}$$

Where *Q* shows the sorption capacity (mg g^−1^), *C*_0_ and *C*_*e*_ are the initial and equilibrium concentration of nitrite (mg L^−1^), *m* is the mass of the adsorbent (mg) and *V* is the volume of the anion solution (mL). According to the results, the maximum sorption capacity (*Q*_*max*_) of NO_2_^−^ was 233 mg g^−1^.

### The reusability of M-Porous IL

**Figure 10:**
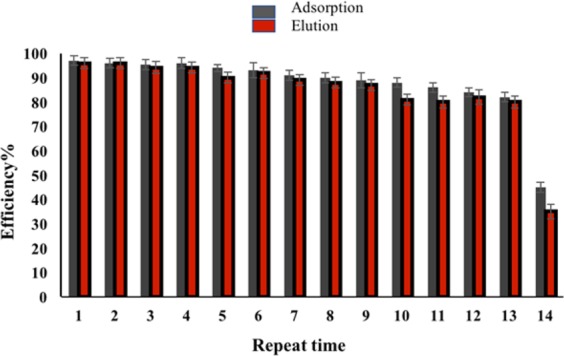
The effect of reuse of M-porous IL in extraction and elution.

Regeneration is one of the important criteria as the cost-effective and large-scale use in real sample analysis^34^. The nanomaterials are displayed in multi-cycles experiments after sorption/desorption operation. The regeneration was taken at the same time during the elution process. The M-porous IL was washed with 1.0 mL of nitric acid and deionized water three times after each DMSPE procedure. The M-porous IL was regenerated into its original functionality and was used for the next run adsorption. The reusability of the sorbent was applied by the efficiency of adsorption and elution (Fig. 10). The M-porous IL adsorbent could be reused 13 times with efficiency of adsorption higher than 80% to the nitrite. Subsequently, the adsorption intensity decreased due to the sorbent wasting during the washing process.

### Interference study

Interference effects of some cations and anions with a relative error of less than ±5% were studied. For interferences such as alkali, alkaline earth cations, Fe (II), Cu (II), Pd (II), Mn (II), Cd (II), Hg (II), and Zn (II), anions such as I^−^, Br^−^, Cl^−^, and CO_3_^2−^, the tolerance ratio was 1000. The tolerance ratio was 700 for SO_4_^2−^ and it was 500 for S^2−^. The results indicated that the foreign materials in the sample solutions had no significant interferences in nitrite separation.

### Analytical performance

Under the optimum conditions described above, the proposed method introduced linearity in the range of 0.1–100 µg L^−1^ with correlation coefficient (R^2^) 0.9996. The limit of detection of NO_2_^−^ was 0.1 µg L^−1^. The inter-day and intra-day relative standard deviations (RSDs) for five independent replicate determinations of 20 µg L^−1^ nitrite were determined to be less than 3.2%. This lower RSD demonstrates the reliability of this method for the determination of nitrite in real samples without considerable matrix effects.

### Analysis of real samples

This method was applied for determining the amount of nitrate and nitrite in water (tap, mineral, and river), fruit (cantaloupe, watermelon, banana, and industrial fruit juice) grains (barley, rice, industrial and traditional breads), legumes (white bean and red bean) and nuts (pistachio and almond). The accuracy of the process was assessed by performing the spike recovery studies. As shown in Tables 1 and 2, the recoveries of the anions varied from 92% to 107% with standard deviation lower than 3.3% for three replicates, thereby indicating the reliability of this method for determination of target anions in real samples without significant matrix effects.

**Table 1:** Determination of nitrite in water samples by proposed method.

Sample	Added (µg L^−1^)		Found (µg L^−1^)		Recovery %		RSD % (n = 3)	
**Water**	NO_2_^−^	NO_3_^−^	NO_2_^−^	NO_3_^−^	NO_2_^−^	NO_3_^−^	NO_2_^−^	NO_3_^−^
Tap	0	0	0.45	2.35	—	—	2.22	2.06
	5.00	5.00	5.41	7.07	99.20	94.40	2.21	2.83
Mineral	0	0	10.57	23.82	—	—	2.34	3.01
	5.00	5.00	15.61	28.59	95.40	100.01	3.77	2.78
River	0	0	63.05	126.32	—	—	3.95	3.86
	30.0	30.0	93.01	155.99	99.86	98.90	3.02	4.01

**Table 2:** Determination of nitrite and nitrate in fruits, grains, legumes and nuts sample by proposed method.

Samples	Added (µg g^−1^)		Found (µg g^−1^)		Recovery		RSD % (n = 3)	
	NO_2_^−^	NO_3_^−^	NO_2_^−^	NO_3_^−^	NO_2_^−^	NO_3_^−^	NO_2_^−^	NO_3_^−^
**Fruits**								
Cantaloupe	0	0	12.91	54.49	—	—	5.43	5.70
	30.0	30.0	42.90	84.07	99.97	98.60	7.69	3.53
Watermelon	0	0	10.34	94.20	—	—	2.14	2.97
	30.0	30.0	40.32	127.17	99.93	109.90	4.71	1.18
Banana	0	0	12.84	84.68	—	—	3.62	2.01
	30.0	30.0	42.81	114.91	99.90	107.67	2.80	0.96
Industrial fruit juice	0	0	17.54	42.67	—	—	1.18	2.85
	30.0	30.0	47.50	72.45	99.87	99.27	2.95	1.38
**Grains**								
Barely	0	0	7.40	22.07	—	—	2.16	3.90
	30.0	30.0	37.41	52.77	100.03	102.33	3.35	2.08
Rice	0	0	6.93	20.85	—	—	1.55	4.13
	30.0	30.0	36.86	50.17	99.77	97.73	4.17	2.60
Industrial Bread	0	0	16.50	41.27	—	—	7.88	4.44
	30.0	30.0	46.01	70.51	98.37	97.47	6.31	3.14
Lavash	0	0	9.60	32.48	—	—	9.04	7.38
	30.0	30.0	39.49	62.25	99.63	99.23	13.17	9.14
Sangak	0	0	4.50	65.38	—	—	7.10	6.62
	30.0	30.0	33.46	96.40	96.53	103.40	10.04	9.11
**Legumes**								
White bean	0	0	6.17	15.59	—	—	3.39	3.66
	30.0	30.0	36.10	45.72	99.77	100.43	5.56	3.43
Red bean	0	0	11.13	25.65	—	—	4.18	4.62
	30.0	30.0	39.01	55.01	92.93	97.87	3.94	3.13
**Nuts**								
Pistachio	0	0	7.24	9.14	—	—	5.65	6.15
	30.0	30.0	37.70	39.52	101.53	101.27	8.81	7.23
Almond	0	0	9.56	14.34	—	—	4.32	5.12
	30.0	30.0	39.41	43.16	99.50	96.07	6.24	7.15

### Comparison with other methods

The determination of nitrite in real samples was compared with some reported methods were listed in Table 3. As shown, the porous IL adsorbent showed several advantages over other sorbents for the determination of nitrite. The limit of detection and RSD % of the proposed method are in some cases lower than others, especially in comparison with expensive and complicated instruments such as ion chromatography and spectrofluorimetry. The reusability of the porous IL sorbent was also compared with other literature. The porous IL adsorbent, in most cases, has high reusability over other sorbents. Comparison of nitrite ion sorption capacities of the different sorbent is listed in Table 3. The amount of nitrite ions adsorbed onto porous IL was greater than that other sorbents, even in comparison with polymeric ionic liquid or porous sorbents. That is because, in addition to the electrostatic interaction between host polymer and guest anion, it is supposed that the presence of pores may contribute to the high sorption capacity toward nitrite anions. Also, the determination of target anion is possible in pH = 4 to pH = 9 and indicates the applicability of porous IL as a sorbent in a wide range of pH. In mentioned researches, various sorbent such as carbonaceous materials, polymers, magnetic nanoparticles and, polymeric ionic liquids immobilized on magnetic nanoparticles or other solid compounds for the speciation and separation of nitrate and nitrite ions are reported. The immobilization of ILs onto solid matrices sometimes requires several steps or various surface functional groups, and in some cases, in the elution step the IL is removed from the solid matrices that reduced sorbent efficiency. In this study, an imidazolium-based ionic liquid was solidified into porous materials and was used directly in the speciation and extraction process. Therefore, the proposed porous material is thus highly efficient and accurate tool for ultra-trace nitrate and nitrite ions detection in various real samples.

**Table 3:** Comparing the current adsorbent with the results of other literature.

Instrument	sorbent	LOD (µg L^−1^)	RSD (%)	Reusability	Operating pH range	Sorption capacity (mg g^-1^)	ref.
Ion Chromatography	MNPs@PIL	0.13	<5	20	8.0	100	^31^
Spectrofluorometry	MMWCNT	34^b^	0.6	12	1.5–11.0	—	^35^
Ion chromatography	SB600	—	—	—	3.0	37.8	^36^
Capillary electrophoresis	—	0.8^a^	6.0	—	2.5	—	^37^
UV-Vis spectroscopy	MGO-Gly	17^b^	1.2	10	4.0–9.0	238	^32^
UV-Vis spectroscopy	Polypyrrole/sawdust	10	<2	1	5.0	—	^38^
UV-Vis spectroscopy	Fe_3_O_4_/SiO_2_/ neocuproine	0.40	4.10	—	5.5	83.3	^29^
UV-Vis spectroscopy	Zein biopolymeric NPs	2.3	0.83,3.8	—	4.0	—	^30^
IS-LME-ET AAS	—	0.013	4.8	—	5.5	—	^39^
ICP-AES	Stable conjugate materials	0.16	3.2	8	5.5	119.2	^40^
Ion chromatography	—	15	8.8	—	—	—	^41^
ICP-AES	Ligand functionalize porous conjugate material	0.45	-	8	3.5	124.3	^34^
UV-Vis spectroscopy	Fe_3_O_4_ nanoparticles	3.1	4.5	—	11.0	—	^42^
Ion-pairing cloud point extraction	—	0.42	4.1	—	4.0	—	^43^
ICP-AES	1-naphthylamine solid conjugate material	0.23	—	8	4.0	135.2	^44^
Fluorescence quenching capillary analysis	—	6.5	4.5	—	1.5	—	^45^
UV-Vis spectroscopy	M-porous IL	0.10	3.2	13	4.0–9.0	233	This work

^a^mg L^−1^.

^b^ng L^−1^.

## Conclusion

Solidification of ionic liquids into porous materials yields porous ionic liquids polymer that integrate the unique characteristics of ILs into porous material and polymers. Due to the abundant positively charged imidazolium groups in the porous IL polymer, it could contribute to an anion exchange procedure. So, this adsorbent is a good candidate for the speciation of nitrite and nitrate anions. Also, porous IL polymer showed a broad operation pH range that makes this polymer to be appropriate adsorbent for the determination of nitrite and nitrate anions in a wide pH range. In comparison to other SPE adsorbent reported in the literature, the new adsorbent, in some cases, showed higher reusability and lower detection limit. In conclusion, this novel magnetic adsorbent is a promising material for the determination of trace amount of pollutants in environmental samples.
